# The Perceived Impact and Usability of a Care Management and Coordination System in Delivering Services to Vulnerable Populations: Mixed Methods Study

**DOI:** 10.2196/24122

**Published:** 2021-03-12

**Authors:** Rubina Rizvi, Courtney VanHouten, Tiffani J Bright, Mollie M McKillop, Shira Alevy, David Brotman, Megan Sands-Lincoln, Jane Snowdon, Barbie J Robinson, Carolyn Staats, Gretchen P Jackson, William J Kassler

**Affiliations:** 1 IBM Watson Health Cambridge, MA United States; 2 Department of Health Services Sonoma County Santa Rosa, CA United States

**Keywords:** vulnerable population, managed care, data integration, advanced technologies, usability, mixed methods study

## Abstract

**Background:**

People with complex needs, such as those experiencing homelessness, require concurrent, seamless support from multiple social service agencies. Sonoma County, California has one of the nation’s largest homeless populations among largely suburban communities. To support client-centered care, the county deployed a Care Management and Coordination System (CMCS). This system comprised the Watson Care Manager (WCM), a front-end system, and Connect 360, which is an integrated data hub that aggregates information from various systems into a single client record.

**Objective:**

The aim of this study is to evaluate the perceived impact and usability of WCM in delivering services to the homeless population in Sonoma County.

**Methods:**

A mixed methods study was conducted to identify ways in which WCM helps to coordinate care. Interviews, observations, and surveys were conducted, and transcripts and field notes were thematically analyzed and directed by a grounded theory approach. Responses to the Technology Acceptance Model survey were analyzed.

**Results:**

A total of 16 participants were interviewed, including WCM users (n=8) and department leadership members (n=8). In total, 3 interdisciplinary team meetings were observed, and 8 WCM users were surveyed. WCM provided a central shared platform where client-related, up-to-date, comprehensive, and reliable information from participating agencies was consolidated. Factors that facilitated WCM use were users’ enthusiasm regarding the tool functionalities, scalability, and agency collaboration. Constraining factors included the suboptimal awareness of care delivery goals and functionality of the system among the community, sensitivities about data sharing and legal requirements, and constrained funding from government and nongovernment organizations. Overall, users found WCM to be a useful tool that was easy to use and helped to enhance performance.

**Conclusions:**

WCM supports the delivery of care to individuals with complex needs. Integration of data and information in a CMCS can facilitate coordinated care. Future research should examine WCM and similar CMCSs in diverse populations and settings.

## Introduction

### Background

Providing comprehensive services to vulnerable populations is a complex task requiring effective and efficient collaboration and resource alignment among various safety net agencies [1,2]. Care management processes are often complicated when such organizations operate in information silos. These processes often involve agencies such as health care, law, housing, and law enforcement, which tend to work independently and lack effective, seamless communication across agencies [3]. To mitigate this growing challenge, synchronization of agencies at different levels is required to successfully provide holistic, client-centered care in an effective and timely manner. These existing silos could be further addressed by using technology as a common platform, which could serve as a reliable information repository for data aggregation, reporting, and exchange. Currently, limited evidence exists about the use, benefits, and shortcomings of social management tools built on advanced technologies.

Sonoma County is a large county in California, with over 500,000 residents [4] and a disproportionately large homeless population compared with similarly sized communities [5]. The 2019 Sonoma County Homeless Census and Survey Comprehensive Report [6] suggested that approximately 3000 homeless people live primarily in vehicles (29%) and emergency shelters (25%); on streets (24%); and in various other shelters (22%), such as tents, transitional housing, or abandoned buildings [6]. This homeless crisis has been intensified by natural disasters, most recently the Kincaid Fire (October 23 to November 6, 2019), which threatened more than 90,000 structures and burned 77,758 acres, resulting in evacuations throughout Sonoma County [7]. This critical situation was recognized by the Sonoma County department leadership and increased the need for a solution that could help address the existing gaps by providing holistic, client-centered care for the county’s residents with complex needs. In 2017, the board of supervisors established a 3-step approach for achieving their objectives by implementing the *Accessing Coordinated Care and Empowering Self-Sufficiency* (ACCESS) [8] initiative to identify the most vulnerable residents and provide them with coordinated services. In efforts to establish a rapid response, the interdepartmental multidisciplinary team (IMDT) was created to coordinate cross-departmental services. The IMDT mainly comprises personnel from each social service department and eligibility specialists, who were hands-on Watson Care Manager (WCM) users, and leaders and executives from programs and services participating in the ACCESS initiative, who engaged indirectly with the WCM system and its output but were not hands-on users. Sonoma County partnered with International Business Machines (IBM) to develop a Care Management and Coordination System (CMCS) to support the IMDT [9].

**Figure 1:**
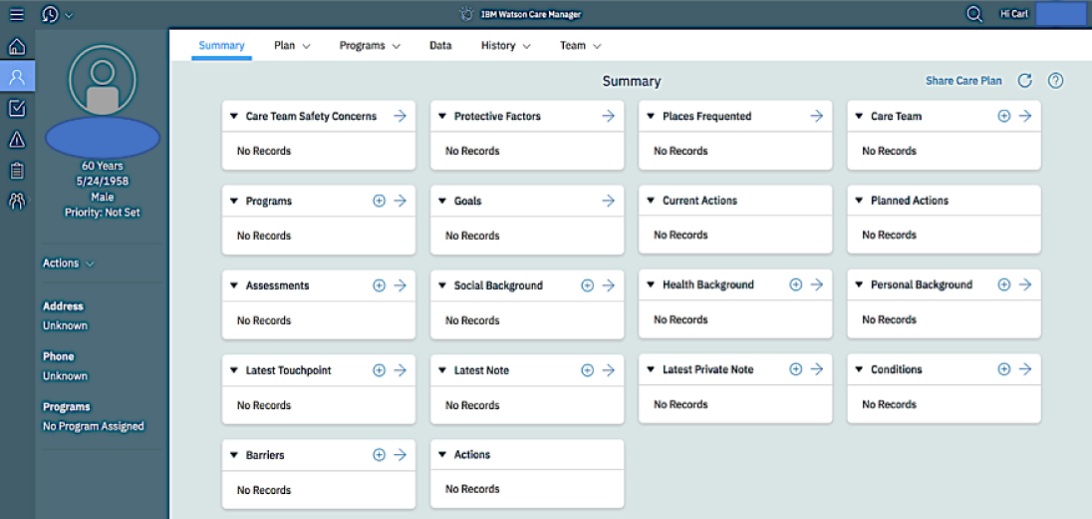
The front-end interface of Watson Care Manager.

The primary objective of the CMCS is to successfully refer vulnerable residents to the services they need most and foster data sharing and collaboration among diverse care professionals to optimize service delivery. The CMCS tool consists of 2 components: Connect360 and WCM. Connect360 is an integrated data hub that receives data from participating agencies and generates a comprehensive, client-specific record as a single data source. These agencies include housing, human services, justice, child support services, substance use disorders, mental health, medical services, aging, and independence. WCM is the front-end interface (our study focus) that displays the consolidated client record to care providers. Using WCM, IMDT members actively enter, aggregate, and render up-to-date information, which enables them to develop integrated care plans for vulnerable clients (Figure 1) [3].

### Objectives

The purpose of this study is to evaluate the user-facing component of WCM to better understand its usability and perceived impact on service delivery processes at the Sonoma County Department of Health and Human Services (SC DHHS). We aimed to examine the role and perceived benefit of using WCM in case management, identify factors facilitating or impeding the use of WCM, and understand user acceptance of WCM with regard to perceived usability and ease of use of WCM.

## Methods

### Study Design

A mixed methods study, including observations, interviews, and surveys, was conducted at SC DHHS in December 2019. Purposive sampling was employed to recruit 2 sets of participants from SC DHHS: (1) WCM end users, which included eligibility specialists, and representatives from various government, law enforcement, and community agencies and (2) department leadership, which included individuals serving in an executive or management capacity.

### Data Collection

The IMDT meetings were observed by four research team members (RR, CV, TB, and MS) with diverse backgrounds. The semistructured interview guides included questions focused on job role, initiation of client contact, client burden of care, client interaction and engagement, WCM use, and interview demographics. The interview guides used for WCM users and department leadership are included in Multimedia Appendices 1 and 2, respectively. Interviews were conducted on-site and over telephone; all were audio recorded, transcribed verbatim, and saved using a Health Insurance Portability and Accountability Act–compliant app Otter (version 2.1.17) [10]. Before each interview, the WCM users completed the Technology Acceptance Model (TAM) [11] survey (Multimedia Appendix 3) to assess the perceived usefulness (PU) and perceived ease of use (PEOU) of WCM.

### Data Analysis

Data were analyzed using thematic analysis based on grounded theory [12]. Coding was performed sequentially on observations and interviews by 4 research team members (RR, CV, TB, and MM). One common transcript from each of the 3 data collection methods (ie, observations, interviews of WCM users, and department leadership interviews) was randomly selected and independently coded to generate an initial set of codes. Coders held 2 adjudication sessions to review codes and achieve consensus. Once a consensus was reached, a final code book for each transcript source, including interviews and observations, was created and applied to the remaining transcripts. Coding reliability was established through consensus among all coders. Dedoose (version 8.3.18) [13] was used to support qualitative data analysis.

### Ethics Statement

All study participants gave written consent to participate in personal audio-recorded interviews. The study was deemed as exempt from human subjects research regulations by the Western Institutional Review Board.

## Results

In total, 3 IMDT meetings were observed; each meeting lasted approximately 3 hours, and field notes were taken by each observer. The WCM tool was used during these meetings, and an example of the summary home page of the front-end interface is shown in Figure 1.

Interviews were conducted with 8 WCM users and 8 department leadership members. Of the 16 interviews, 13 were conducted in person and 3 were conducted over the telephone. Interviews with WCM users lasted for an average of 60 min; the longest interview lasted approximately 130 min. Department leadership interviews averaged approximately 40 min, whereas one leadership interview with 2 individuals lasted for 140 min. The study participant characteristics are aggregated and summarized in Table 1.

**Table 1:** Descriptive characteristics of Watson Care Manager users and department leadership (N=16).

Participants	Value, n (%)	Age (years), range	Age (years), mean (SD)	Gender (female), n (%)	Education (master’s degree or higher), n (%)	Technology skills (intermediate skill level or higher), n (%)
WCM^a^ users (n=8)	8 (50)	31-62	47 (13.2)	6 (75)	5 (63)	7 (88)
Department leadership (n=8)	8 (50)	42-58	51.6 (6.2)	5 (63)	6 (75)	N/A^b^

^a^WCM: Watson Care Manager.

^b^N/A: not applicable.

Analysis of the transcripts resulted in a list of codes. For reporting purposes, we included only the top 10 most frequently applied codes stemming from the analysis of 3 transcript sources (Multimedia Appendix 4).

**Figure 2:**
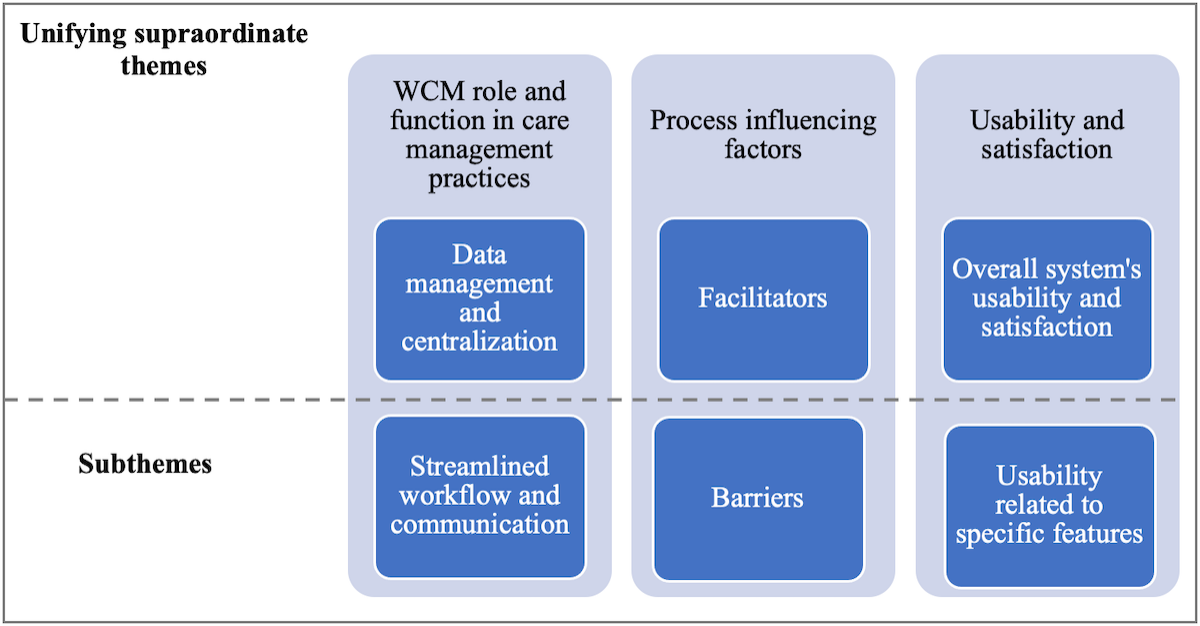
The 3 unifying supraordinate themes and the corresponding subthemes. WCM: Watson Care Manager.

The most granular-level codes were grouped as follows: (1) WCM role and function in care management practices with subthemes of data centralization and streamlined workflows (eg, shared decision making and information aggregation), (2) process influencing factors having subthemes of facilitators and barriers (eg, community demographics and data privacy), and (3) usability and satisfaction with subthemes overall usability and specific tool functionalities (eg, interaction with the interface and alerts; Figure 2).

### Unifying Theme: WCM Role and Function in Care Management Practices

Observations of IMDT meetings consistently suggested that WCM was used as the main tool for data review, both by leaders and users. WCM provided IMDT team members with an opportunity to have a collaborative team discussion about a client’s status and review the data before the IMDT meeting. Despite its advantages, it was observed that 2 WCM users continued to reference paper copies of case information as a supplemental resource for facilitating updates and discussion.

Slight differences were observed between department leadership and WCM users. Leadership primarily discussed the positive impact WCM had on employees and department operations, whereas users tended to discuss the impact of WCM in the context of facilitating daily task accomplishments, establishing client rapport, and strengthening existing relationships. Despite these differences, these interviews revealed a praxis-based approach whereby descriptions of task achievement were used to illustrate how WCM supported larger goals. Analysis of all the observations and interviews revealed 2 principal themes of use: (1) data management and centralization and (2) streamlined agency communication.

#### Subtheme: Data Management and Centralization

Both program leaders and hands-on users repeatedly referenced the role of WCM as a data management tool to centralize client information and serve as a platform to reliably retrieve and store client information. In addition to progress-related updates, WCM helped users to stay up to date on short-term, practical tasks such as taking clients to probation or court hearings, delivering public transit vouchers, or keeping medical follow-up appointments. The following excerpt from the field notes validated how WCM facilitated scheduling a client’s appointment, thereby enhancing task performance efficiency:

During a client presentation, the case manager requested a new medical appointment, and the nurse made the medical appointment instantly. The appointment was scheduled before the conversation was even over.

Observation note

WCM users routinely retrieved and documented case notes in up to 7 different systems simultaneously before working in WCM. Furthermore, documentation of client interactions in multiple systems was necessary to meet state and federal reporting requirements; however, they did not have a platform to store or share case-specific information such as care plans or client goals:

If Watson didn’t provide that service [providing a platform across multiple systems], I’m doing what I’ve been doing that is, I have seven systems, and I work in those seven systems to gather information.

Participant #6, user

We also don’t have a place where our care plan is currently living. And so Watson is the only place that the care plan actually lives. The living document. So, I think that is really important.

Participant #6, user

I use it to go in and see what one of the other team members have [already completed] when there is something that needs to be done. To make sure that anything that needs to be done for the benefit of the client is being done.

Participant #4, user

WCM not only helped in care delivery but also helped users maintain their client’s enrollment in government support programs through an *alerts* feature. Department leadership noted that securing and maintaining such support is integral to helping a client achieve stability but that it can sometimes be a burden on client managers whose clients are often enrolled in several programs with different requirements and renewal terms:

Well, I kind of go back to what I was saying about the alerts. If I missed the alert, and I didn't reach out to the client and inform the client this is upcoming [and] this has to be done, or you will lose the benefit [and]to go through the process of trying to get that benefit reestablished is a lengthy process. So, it helps the client, and it helps me to know that I'm doing my job.

Participant #4, user

In addition, by aggregating client eligibility information, WCM coordinated multiple application processes, allowing the client to apply for multiple programs at one time. This coordination reduced the cognitive burden on employees and helped reduce unnecessary client effort by avoiding the need to constantly obtain required documentation or navigate multiple application procedures:

A lot of [applications and eligibility] interactions we use Watson Care Manager. We can like coordinate to get verification before my interview [with the client]. So, at the time of the interview begins or the time I meet the client like they can already have everything ready so it’s like “here you go. Like I have it and it's more straightforward my end that I can approve them all one time and just collect the application.”

Participant #2, user

Department leadership interviews echoed the resulting benefits of WCM by rendering data that are streamlined and centralized, serving as a one-stop shop to learn about the client’s current status and to plan out the next steps:

That’s one of the things that everybody has a great help for with Watson, how we use that information, bringing our respective data together, and work in a more coordinated fashion.

Participant #15, department leadership

#### Subtheme: Streamlined Workflow and Communication

Streamlined workflow and communication emerged as common subthemes, from interviews of both leaders and hands-on users. WCM played an essential role in effective and efficient communication not only within the agency but also between other agencies. More specifically, agencies related to health care, housing, and justice were able to communicate across silos to address time-sensitive client needs. Examples included understanding a client’s current health status and making timely health care appointments, ascertaining the availability of housing spaces based on individual needs, and verifying the parole status of the client. Observations noted up-to-date exchange of information between WCM users from different agencies, resulting in a comprehensive, timely care delivery plan that prioritizes clients’ needs and avoids potential adverse repercussions:

Client released from jail to the crisis residential unit and needs mental health services but refuses. Group discussion around options, pathways – how to get client to seek mental health. Probation officer reminded the criticality of client’s compliance with receiving mental health treatment since it was one of the conditions of his parole.

Observation note

During interviews, both WCM users and department leadership discussed how WCM use and IMDT meetings facilitated real-time discussions among the pertinent care providers from the representative agency, enabling more informed, shared, and efficient decision making. Both users and department leadership highlighted specific descriptions of how case managers and other client specialists were able to advocate for their clients. Users also shared their prior experiences before the introduction of WCM and described operating in silos and on different information technology systems. They described the benefit of provisioning a holistic, client-centered approach with a common shared platform with specific case examples:

We could all talk about how the client is doing within the shelter. And so the [parole officer and shelter manager] worked with her on a plan...and everything got better from there. We can have a shared understanding.

Participant #6, user

Effects on communication and coordination led to changes in workflow processes, and they were described by one user as follows:

A lot of [applications and eligibility] interactions we use Watson Care Manager. We can like coordinate to get verification before my interview [with the client]. So, at the time of the interview begins or the time I meet the client like they can already have everything ready so it’s like, here you go. Like I have it and it's more straightforward my end that I can approve them all one time and just collect the application.

Participant #2, user

In addition to integrating data and alerts from multiple agencies, WCM facilitated cross-program collaboration by providing a shared vocabulary. This common language and shared context fostered mutual understanding among agencies:

I mean we've spent many hours talking about like really silly things but like things that are weirdly fundamental for, like, one government agency talking to another like, what does date of admission mean? What does it mean to the jail, what does it mean to behavioral health? Are we tracking the right date of admission?... oh my god what a headache to have a conversation but at the same time, like us, understanding that one person's definition of this thing is radically different from someone else's is important.

Participant #6, user

### Unifying Theme: Process Influencing Factors

Facilitators and barriers influencing the implementation and use of WCM were primarily identified through participant interviews from both executives and the hands-on users of WCM. During interviews, factors that shaped engagement with WCM were described.

#### Subtheme: Facilitators Influencing WCM Implementation and Use

WCM users from the IMDT and department leadership discussed opportunities to expand WCM to additional groups of clients in other US counties, apart from Sonoma County. The IMDT attendees acknowledged the benefits and value of WCM and provided examples of how performance improvement enabled client success.

...so, it helps the client, and it helps me to know that I'm doing my job.

Participant #4, user

It's just everything's there in your fingertips and you just might have to scroll down a little bit, but everything there...my success rate is so much more...ultimately that's what matters so like improving care, [I am] pretty satisfied with it.

Participant #2, user

Department leadership described their viewpoint on the role of WCM in facilitating a collaborative environment. It was valuable in helping their transformation toward a client-centered culture. They also discussed WCM’s flexibility, which enabled diverse departmental participation and improved care coordination. Multiple agencies recognized the value of WCM’s role in streamlining care delivery and facilitating efficiency. Finally, this environment indicated a strong and widespread commitment to collaboration and willingness to participate in WCM use:

Our ability to show that [WCM] is effective helps us advocate for and leverage your resources. If they see it as successful then they are willing to put more resources into it.

Participant #15, department leadership

This work that you're [WCM] doing, and what has been accomplished thus far with Watson - I think is groundbreaking, and really has great potential to go to scale.

Participant #15, department leadership

In addition, users and department leadership highlighted the unique aspects of Sonoma County that may have contributed to WCM’s success in their client population. Notably, the manageable, medium-sized county with active community-based agency participation facilitated and maximized WCM’s utility and resulting outcomes:

I think Sonoma is in a very good place in that we're medium sized county and we, we have a lot of community-based organizations that work with us are already under contract and so it's probably more possible in Sonoma County than anywhere to be successful.

Participant #9, department leadership

#### Subtheme: Barriers Influencing WCM Implementation and Use

Currently, there is a lack of awareness and knowledge about how the ACCESS initiative operates. Barriers exist and impede its widespread adoption across other counties within California:

...so I think we have work to do, and a lot of that will be about outreach!

Participant #9, department leadership

The concerns around data privacy and security and the complexity of legal requirements also present implementation challenges. Although leadership expressed the desire to participate in the ACCESS initiative, they expressed concerns about meeting strict federal and state reporting requirements on data sharing in addition to their struggle in identifying appropriate funding streams:

You know, we have these really, really, strict policies on our data, so we’ve been able to do some match where we’ve been able to get some cohort data and then match it against our system, but we haven’t been able to into the universal system and you know that’s been a little frustrating.

Participant #9, department leadership

In user interviews, participants discussed the possibilities of using WCM for simple data organization and commented that they had fewer data systems and necessary log-in requirements:

I have like nine systems...that I use on a frequent basis. And then I have to use the additional two or three, four programs...So, with that and then I have to use Watson Care Manager because there's not much integration with that so I have to do three different systems with my case notes, so you have three different IDs and passwords you have to log in and I have like 12.

Participant #2, user

One user, however, voiced concern about duplicate efforts and manual tasks required when WCM was not able to retrieve all client-related data from various resources. They suggested that improved integration was needed to make the processes more efficient:

So, while at the moment it isn't frustrating or feeling like an extra piece of work...it's just the system is not set up right now.

Participant #3, user

### Unifying Theme: Usability and Satisfaction (Users’ Perspective)

Users’ perception of WCM was analyzed based on data from observations, user interviews, and surveys. Perspectives on usability, including both PU and ease of use, and satisfaction are presented in Table 2.

**Table 2:** Responses from the Technology Acceptance Model survey.

Variable and TAM^a^ survey questions^b^		Reported TAM score, median (minimum-maximum)
**PU^c^**		
	Enables me to accomplish tasks more quickly than other products	4 (3-7)
	Improves my job performance	5 (3-7)
	Increases my productivity	4.5 (2-7)
	Enhances my effectiveness on the job	4.5 (3-7)
	Makes it easier to do my job	4.5 (3-7)
	I have found WCM^d^ useful in my job	5 (3-7)
Median PU		5 (4-5)
**PEOU^e^**		
	Learning to operate was easy	4.5 (2-7)
	Easy to get to do what I want it to do	5 (3-6)
	Interaction is clear and understandable	5 (3-7)
	Flexible to interact with	5.5 (2-7)
	Easy for me to become skillful	6 (2-7)
	I found WCM easy to use	5 (2-7)
Median PEOU		5 (4.5-6)
Overall combined median		5 (4-6)

^a^TAM: Technology Acceptance Model.

^b^The Technology Acceptance Model version used in this study had 12 questions, 6 assessing perceived usefulness and 6 assessing perceived ease of use, and they were scored on a 7-point Likert scale where 1=extremely disagree and 7=extremely agree.

^c^PU: perceived usefulness.

^d^WCM: Watson Care Manager.

^e^PEOU: perceived ease of use.

#### Subtheme: Overall Perceived Usability and Satisfaction

Overall, WCM users found the system to be well suited for their needs, serving as a one-stop shop for users by providing them with client-related data under one coherent platform. Users described WCM as being able to reliably house client information and provide easy access to relevant, time-sensitive information. Most importantly, WCM did not simply replicate previously existing electronic or paper forms but dynamically supported client care delivery by providing access to the latest data on client status and progress-related updates. Interviews also indicated the agility of WCM in allowing for rapid customization and the creation of a tool personalized according to their preferences.

TAM surveys were completed by WCM users and, on average, took 10 to 15 min (Table 2).

User feedback also suggested that WCM was not being used to its full potential and that specific WCM features were underused. WCM users suggested that additional individualized training might allow for better optimization of WCM capabilities:

...it's almost like having a Ferrari, and only knowing how to drive a Ford.

Participant #4, user

#### Subtheme: Usability Related to Specific Features

The *alerts* feature was one characteristic of the tool that was valuable to WCM users. A benefits coordinator used the alerts feature to notify managers about programs for which their client was eligible, to set reminders for application deadlines, or to notify regarding benefits renewal dates. Users described the alerts as valuable reminders that helped them monitor and maintain their clients’ enrollment and avoid coverage gaps. Department leadership interviews also expressed agreement on the benefits of the alert feature. Leadership noted that securing and maintaining such support is integral to helping a client achieve stability but is often a burden on client managers whose clients are often enrolled in several programs with different requirements and renewal terms:

Oh, yeah, I have one thing that I can put up as why Watson Care Managers are so needed is to share the information piece, and the alerts, put on that front page of the summary page.

Participant #2, user

Users also mentioned 2 other features that they thought were useful. Both the summary (with demographics) page and the availability of a clients’ picture facilitated with their tasks by providing succinct, identifiable, and useful data:

The one thing that I think is the most useful is when you look at is the profile. The first thing is that demographics that picture. The name, the date of birth, the address and the phone number. That is my everything I minded the most useful.

Participant #2, user

Findings from observations, interviews, and the TAM survey indicated that users found WCM to be well suited for their needs. A high level of PU (PU=5) and PEOU (PEOU=5) was indicated as a result of the TAM survey, and WCM was considered a reliable and useful tool that was easy to use and helped improve participants’ overall productivity.

It should be noted that some WCM users indicated concern about the lack of interface optimization, such as unnecessary clicking and scrolling. The few users that reported this were not familiar with the system, which influenced their ability to efficiently perform tasks. This feedback was further supported by the median TAM score of 4 (with a minimum-maximum value of 3-7), especially for the responses around *accomplishing tasks quicker*:

Part of it's about learning to use the system and part of it's about minimizing clicks...

Participant #6, user

## Discussion

### Principal Findings

Provision of effective, holistic, client-centered care for vulnerable populations presents a complex challenge requiring substantial collaboration and careful coordination. This study provides unique insights into how a technology solution serves as an essential anchoring tool in a case management model. It enables data consolidation across agencies under one platform and serves as a common communication channel. The tool cultivated an environment of shared decision making and strengthened relationships among agencies.

The CMCS data hub, comprising WCM and Connect360, helped to retrieve and consolidate data in one shared place. It provided case workers with most recent data, such as court dates, benefit eligibility due dates, and doctors’ appointments for clients who often need timely actions. According to users, WCM played a valuable role in addressing the challenges unique to social work with homeless populations where information is often transient, including mobile phone numbers, addresses, and contact information. WCM users and department leadership also provided feedback on barriers, including lack of funding, complex reporting structures, and limited awareness about the ACCESS initiative. Despite these barriers, WCM was perceived as a useful and easy-to-use tool associated with high user satisfaction, as validated by observations, interviews, and TAM responses.

The WCM tool was recognized for its high level of usability and satisfaction. Key features included alerts, information summary page with demographics, the client picture, and the client list page, which served as an index to pull all the client records stratified by priority status. The overall TAM score results and the scores across the 2 dimensions of usefulness and ease of use reinforced findings from the qualitative analysis.

Furthermore, the TAM survey results may suggest a greater likelihood of future use of the WCM tool, consistent with previous literature [14]. No association was observed between the level of interaction, education, and self-perceived skills in the technology when comparing these variables with TAM results (Table 2), which is informative of its high usability as scored consistently across diverse sets of users.

### Limitations

Although these findings illustrate the capabilities of an advanced technology-based tool (WCM) in enhancing the care delivery processes within a county’s homeless population, the study is not without limitations. These findings focused only on one specific cohort of vulnerable, homeless individuals residing in a single county. We also did not objectively measure the impact of the tool on long-term client outcomes. Instead, the study examined the perceived and observed usability and effects on workflow from the perspectives of program leaders and hands-on users. We did not incorporate client perspectives in this study.

### Conclusions

This mixed methods study provides a better understanding of the public health impact that social care management tools such as WCM may have on counties in need of care coordination. Future research investigating the impact of WCM on outcomes (eg, social, clinical, and economical) and the value of WCM for additional communities and diverse subpopulations is essential to build upon these existing findings.

## Appendix

Multimedia Appendix 1 Interview guide for Watson Care Manager users.

Multimedia Appendix 2 Interview guide for department leadership.

Multimedia Appendix 3 Technology Acceptance Model.

Multimedia Appendix 4 Top 10 frequently applied codes stemmed from the analysis of 3 sources of transcripts. ACCESS: Accessing Coordinated Care and Empowering Self-Sufficiency WCM: Watson Care Manager WPC: Whole Person Care.

## Abbreviations

ACCESS: Accessing Coordinated Care and Empowering Self-Sufficiency
CMCS: Care Management and Coordination System
IBM: International Business Machines
IMDT: interdepartmental multidisciplinary team
PEOU: perceived ease of use
PU: perceived usefulness
SC DHHS: Sonoma County Department of Health and Human Services
TAM: Technology Acceptance Model
WCM: Watson Care Manager
