# Supplementary material for: The Perceived Impact and Usability of a Care Management and Coordination System in Delivering Services to Vulnerable Populations: Mixed Methods Study
Source: J Med Internet Res. 2021 Mar 12;23(3):e24122. doi: 10.2196/24122 (PMC7998322; doi:10.2196/24122)
Supplement: Multimedia Appendix 1 [file jmir_v23i3e24122_app1.pdf]

APPENDIX 1: Interview Guide for WCM Users

Thank you for agreeing to be interviewed today. We are part of a research team that is assessing the perceived usability; user experiences and impact of integration of IBM care management and coordination system (WCM) into client care management workflows.

The interview will last between 30 minutes and one hour. Before we get started, we'd like to have you sign a consent form stating that you are participating voluntarily. It also states that this interview will be completely confidential, and your name will not be used in any kind of reporting or publishing. We will combine the results from all of our interviews to provide a better picture of the perspectives and experiences of care managers who use WCM in order to improve product usability and make recommendations to strengthen client-centered care management approaches for vulnerable populations. We would like to tape-record the interview, on IBM computer that is HIPAA-compliant, if that is alright with you. It will not be linked to your name in any way; but it will be transcribed, with any identifying information removed, so that the research team can better analyze the data.

Is that okay? Do you have any questions? Ok, let's get started.

Interview Guide

**IBM Interviewer**	
**Participant ID**	
Date	
Start time	
End Time	

A. General Questions:

1. Job role overview and experience with WFO

- Can you please tell me more about your job title, role, responsibilities and how long have you been in this role?
- About how long have you been using WCM?

2. Performance

- How do you know when you're doing a good job in your current role?
- To your knowledge, is your department measuring performance for individual care managers? If yes, how and what is measured?

3. Client related information

- Can you please tell me more about the client load in terms of how many clients/individuals are assigned in total, how many clients/individuals do you see on a given week/day?
- Can you please tell me how clients are assigned?
- What are some of the reasons clients switch between case managers? Does this happen frequently? Who initiates the request?

B. Workflow processes before and after WCM integration:

In next 2 sections, we would like to learn more about your workflow processes and related task both before and after WCM integration.

1. ***Preparing for the first interaction with the client***
   - How do you prepare for your first interaction with the client? Does preparation vary for different clients? Tell me about that.
   - What type of information do you already have about your clients when you are first assigned?
   - Can you tell me about the different channels available to receive a client referral (e.g., phone, email, database)? What are the most common ways you get connected to new clients? Any change after WCM integration?
   - What information do you gather during this interaction? Are there logs or recordings? Where is this information stored?

2. Care management, coordination system and use of other tools
- Let's say I am a resident of Sonoma County who just became homeless and qualifies for intensive case management through the ACCESS initiative. What would be the first thing that would happen to engage me from your point of view? Can you walk me through the process of engagement, interaction, and follow-up? First go through the process before WCM integration (if you have worked more than 2 years) and then inquire what happened after WCM
- What are the strengths (benefits, enablers) and weaknesses (road blocks, challenges) associated with each workflow in terms of time and productivity, client care, care coordination)?
- IBM Connect 360 integrates 4 systems to create a master data record for each client from: CalWIN for eligibility operations; SWITS for drug and alcohol dependency; Avatar for mental health; and IJS for proprietary justice data. Given your role as a care manager or ______, do you rely on any other systems outside of WCM? If yes, how many systems do you work with and what are they?

C. WCM usage and training
- Do all SC Cases Managers complete dashboard sections equally?
- On average, how frequently do you engage with WCM per day/week/month?
- Have you received any training, by whom? How long?
- Would training on WCM have been helpful? Why or why not?
- Do you use WCM outside of the IMDT? If yes, how and why?

D. WCM interface
- Do you directly interact with the WCM? If Yes What computes system do use to interact with WCM?
- Can you please walk me through the process to open, close and re-open cases?
- Does WCM help with pathways/decision points? Like determining the 'best facility' for a client? Or where a client is eligible? Like Turning Point> Fact> Whole Person Care>
- What are the main strengths and weaknesses of the interface?
- What specific features/functionality of WCM do you find the most helpful/used the most? Least helpful/used least?
- What happens if you see the alerts? Ask about ability to clear alerts
- What feature you use the most? Please rank them in the order you use them
- How easy or difficult was it to learn how to use WCM?
- Does WCM include a section like FAQ or frequently used resources?

- Can WCM connect with other systems to provide additional details about protective orders to better protect clients?
- Is there anything you want to add/ remove from WCM (design and/or functionality) ?
- What are your thoughts about WCM being used in other CA counties to allow for easier data sharing about clients who may have lived in or been displaced from another county? Benefits? Drawbacks?

E. Use of WCM and the resulting outcomes (Client well-being and interdepartmental connectedness) (~ allocated time=8 mins)

- From your perspective, how would you assess or evaluate whether a client/individual's needs are being well managed?
- Can you describe whether WCM is enabling you to provide better care coordination and management?
- Can you tell me about a time when you were able to use the information you learned from WCM or with the IMDT to change a course of action or decide to take no action with a client? (non-events)
- Have you noticed a difference in your ability to collaborate more effectively with other Sonoma County departments since using WCM?

F. Usability and Satisfaction

- Thinking back, how satisfied were you with the previous way of doing things i.e., before WCM? (On a scale of 1-5 with 1 being least and 5 being most satisfied)
- How satisfied are you now after integration of WCM (if you have been here for more than 2 years) into the care management process? (On a scale of 1-5 with 1 being least and 5 being most satisfied)
- Looking back to the time before you started using WCM, what expectations did you have about it?
- In terms of efficiency and productivity clinical workflow, client care, client outcomes, client satisfaction, staff/team interaction
- Has WCM met your expectations? Why or why not?
- Is there anything else that you'd like to tell me about WCM that I haven't asked?

G. Demographic/professional information (~ allocated time=2 mins)

- What is your age?
- What is your educational background? What year did you graduate from your most recent educational program?
- How would you rate your level of comfort with technology in general – novice (beginner), intermediate, or expert?

That's all the questions I have. Do you have any questions for me? Thank you for taking the time to talk with me today – we really appreciate hearing your perspectives!
