# Supplementary material for: The Perceived Impact and Usability of a Care Management and Coordination System in Delivering Services to Vulnerable Populations: Mixed Methods Study
Source: J Med Internet Res. 2021 Mar 12;23(3):e24122. doi: 10.2196/24122 (PMC7998322; doi:10.2196/24122)
Supplement: Multimedia Appendix 2 [file jmir_v23i3e24122_app2.pdf]

APPENDIX 2: Interview Guide for Department Leadership

Thank you for agreeing to be interviewed today. We are part of a research team that is assessing the use and impact of IBM Watson Care Manager (WCM) on care management workflows, operational efficiency, and collaboration among diverse care professionals to optimize service delivery.

The interview will last between 30 minutes and one hour. Before we get started, We would like to have you sign a consent form stating that you are participating voluntarily. It also states that this interview will be completely confidential and your name will not be used in any kind of reporting or publishing. We will combine the results from all of our interviews to provide a better picture of the perspectives and experiences of Sonoma County stakeholders (e.g., executives and directors) who lead departments participating in the ACCESS initiative. Results from this study will be used to generate recommendations for improvements to WCM and to strengthen client-centered care management approaches for vulnerable populations. We would like to tape-record the interview, if that is alright with you. It will not be linked to your name in any way; but it will be transcribed, with any identifying information removed, so that the research team can better analyze the data.

Is that okay? Do you have any questions? Ok, let's get started.

Interview Guide

IBM Interviewer	
Participant ID	
Date	
Start time	
End Time	

A. Professional role overview

- What is your job title and professional role here in Sonoma County? How long have you been in this role?
- What are the major responsibilities of your position?
- What does a "typical" day at work look like for you?
- Approximately how many staff do you currently supervise?

B. Department description

- What is the mission and vision of the Sonoma County department you oversee/lead?
- What programs and/or services does your department provides for Sonoma County?
- What would you say are the major challenges facing your department at this time?

C. Client's burden of care =

- Can you tell me about the most pressing challenges your clients face?
- How does your department help clients address those challenges?
- What systems/processes do clients need to navigate in order to access services your department provides?

- What barriers exist that can make it difficult for your clients to access and/or maintain access to such services?
- What enablers exist that can facilitate your clients' access to these services?

D. Departmental involvement with ACCESS initiative

- Can you tell me a little bit about your department's involvement with the ACCESS initiative, to date? What's been working well? What challenges has your department experienced?
- From your perspective, what has the impact of participation in the ACCESS initiative been to date?

E. Perspectives on IMDT approach and role of WCM

- How many of your department's staff are currently assigned to the ACCESS initiative's IMDT? Why were these particular staff selected?
- What do you know or have heard about IBM's Connect 360/WCM and its use in the IMDT setting for client case management?
- From your perspective, what do you believe the value or benefits are of the IMDT-based approach to client-centered care management for clients? For participating staff?
- From your perspective, what do you think the challenges are of the IMDT-based approach for clients? For participating staff?
- Do you ever interact with WCM? How?

F. Client well-being and interdepartmental connectedness

- From your perspective, how would you assess or evaluate whether a client/individual's needs are being well managed?
- From your perspective, what do you look for in terms of signs of improvement in client well-being or stability?
- Have you noticed a difference in your department's ability to collaborate more effectively with other Sonoma County departments since staff have started participating in the IMDT and are using WCM?

G. Demographic/professional information

- What is your age?
- What is your educational background? What year did you graduate from your most recent professional program?
- How would you rate your level of comfort with technology in general – novice (beginner), intermediate, or expert? Provided them with the definition of each level.

That's all the questions I have. Do you have any questions for me? Thank you for taking the time to talk with me today – we really appreciate hearing your perspectives
