# Supplementary material for: The Perceived Impact and Usability of a Care Management and Coordination System in Delivering Services to Vulnerable Populations: Mixed Methods Study
Source: J Med Internet Res. 2021 Mar 12;23(3):e24122. doi: 10.2196/24122 (PMC7998322; doi:10.2196/24122)
Supplement: Multimedia Appendix 3 [file jmir_v23i3e24122_app3.pdf]

APPENDIX 3: Technology Acceptance Model (TAM)

The TAM version used in this study had 12 questions; six assessing PU and six assessing PEOU, and were scored on a 7 points Likert scale where 1= extremely disagree and 7=extremely agree). Survey responses to the TAM were analyzed with descriptive statistics, and median, minimum and maximum values were calculated for all responses.

	**1**	**2**	**3**	**4**	**5**	**6**	**7**
1. Using WCM in my job enables me to accomplish tasks more quickly than other products in its class.							
2. Using WCM improves my job performance.							
3. Using WCM in my job increases my productivity.							
4. Using WCM enhances my effectiveness on the job.							
5. Using WCM makes it easier to do my job.							
6. I have found WCM useful in my job.							
7. Learning to operate WCM was easy for me.							
8. I found it easy to get WCM to do what I want it to do.							
9. My interaction with WCM has been clear and understandable.							
10. I found WCM to be flexible to interact with.							
11. It was easy for me to become skillful at using WCM.							
12. I found WCM easy to use.							
