# Supplementary figures and images for: The Perceived Impact and Usability of a Care Management and Coordination System in Delivering Services to Vulnerable Populations: Mixed Methods Study
Source: J Med Internet Res. 2021 Mar 12;23(3):e24122. doi: 10.2196/24122 (PMC7998322; doi:10.2196/24122)

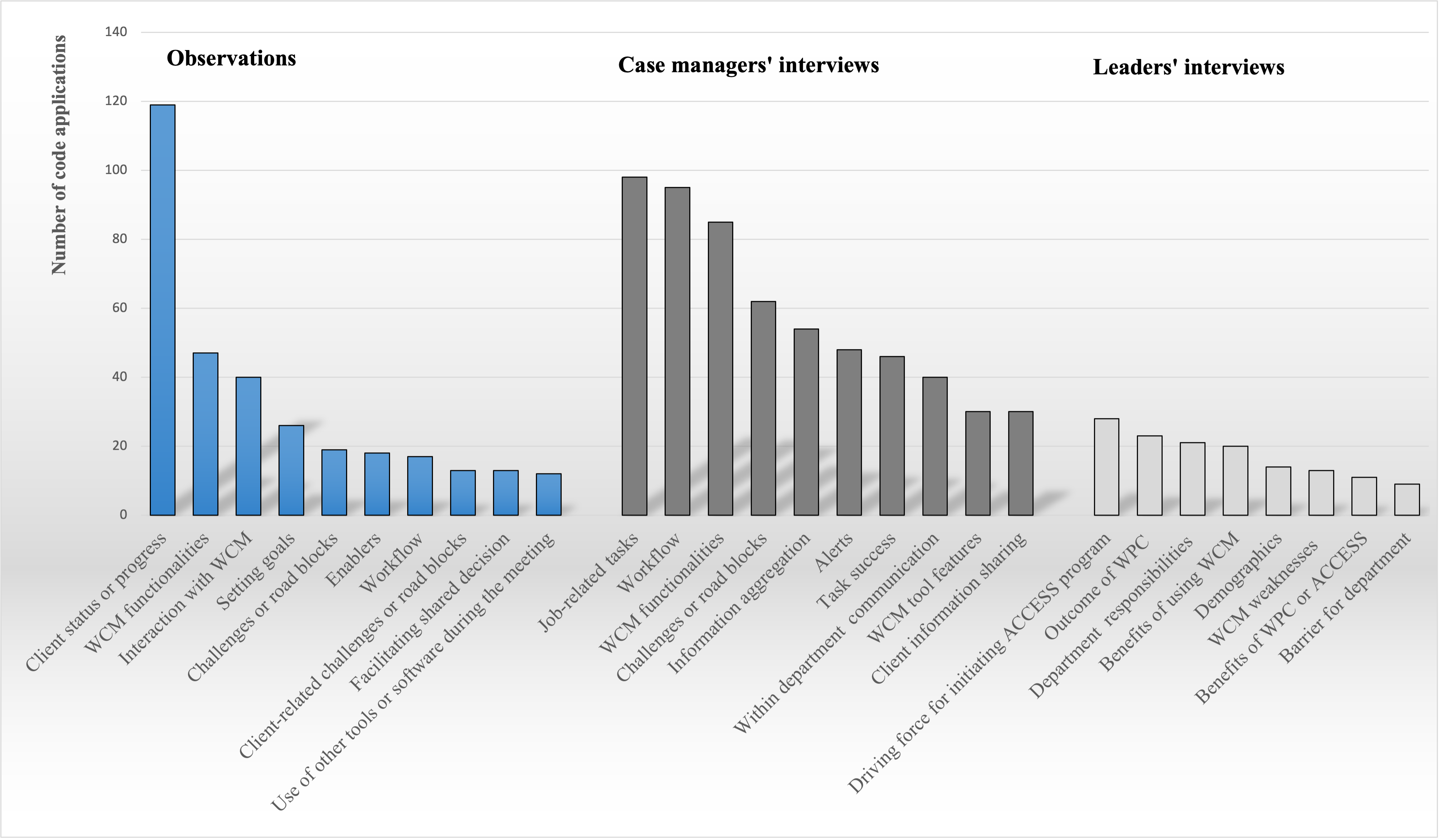

Supplement: Multimedia Appendix 4 [file jmir_v23i3e24122_app4.png]
